# Antimicrobial Use in Extensive Smallholder Livestock Farming Systems in Ethiopia: Knowledge, Attitudes, and Practices of Livestock Keepers

**DOI:** 10.3389/fvets.2020.00055

**Published:** 2020-02-26

**Authors:** Biruk Alemu Gemeda, Kebede Amenu, Ulf Magnusson, Ian Dohoo, Gunilla Ström Hallenberg, Gezahegn Alemayehu, Hiwot Desta, Barbara Wieland

**Affiliations:** ^1^Animal and Human Health Research Program, International Livestock Research Institute (ILRI), Addis Ababa, Ethiopia; ^2^Department of Microbiology, Immunology and Veterinary Public Health, College of Veterinary Medicine and Agriculture, Addis Ababa University, Bishoftu, Ethiopia; ^3^Department of Clinical Sciences, Swedish University of Agricultural Sciences, Uppsala, Sweden; ^4^Department of Health Management, Atlantic Veterinary College, University of Prince Edward Island, Charlottetown, PE, Canada

**Keywords:** antimicrobial use, livestock, smallholders, knowledge, attitude

## Abstract

Antimicrobial resistance (AMR) is a major public health threat, and inappropriate antimicrobial use (AMU) in food animal production can contribute to the global burden of AMR in humans. This study was conducted to understand knowledge, attitude, and practice (KAP) of smallholder livestock owners regarding antimicrobial use, residue, and resistance in three agro-ecological zones and production systems in Ethiopia. A cross-sectional study based on structured interviews was conducted. Twenty-one items were used to assess farmers' KAP. Item response theory (IRT) model and Cronbach's alpha were used to assess the KAP measurement scales. Inferential analyses were used to compare the differences in the practices in terms of the farm and socio-economic characteristics. There was a difference in the type of antimicrobials reported use between agro-ecological zones and production systems. Pastoralists most commonly used antibiotics (86.7%) followed by anthelminthics (70.8%). Overall, tetracyclines (36.4%), aminoglycosides (31.3%), and trimethoprim-sulfonamides (6.2%) were the most frequently used classes of antibiotics across the study sites. Human preparation antibiotics (tetracyclines) were also being used for veterinary purposes by 18.5% of pastoralist households. About 81.6% of livestock owners surveyed reported to have access to veterinary drugs although access varied between agro-ecological zones and production system. About 72.3% of pastoralists administered antibiotics by not following through the full treatment course. Moreover, 70% of respondents were not aware of the recommended withdrawal periods of milk and meat after antibiotic treatment. It was noticed that around 80 and 70% of respondents had a tendency to give doses higher or lower than recommended of antimicrobials, respectively. The study confirms the need for interventions to increase knowledge among smallholder farmers to improve the way antimicrobials in general and antibiotics in particular are used in these settings. In addition, professional involvement, supervision, and guidance can also lead to more efficient antimicrobial use by smallholder livestock owners. The study also highlights the need for research into the development of usable tools that measure antibiotic knowledge and attitudes.

## Introduction

Antimicrobials are applied in livestock farming for number purposes such as therapeutic (treating sick animals), metaphylaxis (control treatment of whole herd in case of disease outbreak), prophylaxis (preventive treatment), and growth promotion (1). The increasing demand for animal protein especially in developing parts of the world is causing an increase in animal production, and in connection with this, antimicrobial use in food-animal production was estimated to rise by 67% between 2010 and 2030 (2). Apart from the historical and the current positive contribution of antimicrobial use in animal health and production management, there exist a number of possible drawbacks associated with the use of antimicrobials in food-animals. Mis(use) of antimicrobials in food animals is potentially causing the emergence of antimicrobial-resistant bacteria strains by increasing selection pressure on bacteria to become resistant (2, 3). Other negative consequences associated with antimicrobial use in food animals is the occurrence of unacceptable level of drug residues in food of animal origin. The inappropriate use of antimicrobials in food animals can result in accumulation of toxic and harmful residues in animal products that can further affect the health of consumers largely by causing allergic reactions (4, 5). Therefore, the antimicrobial usage in food animals is indeed becoming a global issue associated with food safety and public health.

The growing concern regarding emergence of bacteria resistant to antimicrobials and their potential for transmission to humans via animal production has led various authorities worldwide to implement measures to decrease antimicrobial use in livestock production (6–9). Though some studies indicate the occurrence of naturally resistant bacteria, the substantial use of antimicrobial agents in animal production is suspected as one of the important factors driving the emergence of antimicrobial resistance in bacterial strains (10–12). Antimicrobial resistance is a major public health crisis (13, 14), threatening the return of untreatable infections and deaths on a massive scale if appropriate actions are not taken (15). To reduce the problem of human infections caused by resistant bacteria transferred from animals, there is continuous pressure to restrict the use of antimicrobials in animals (7, 9).

Apart from the public health impact, an increasing prevalence of antimicrobial resistance, particularly to frequently used antimicrobials in livestock, could also lead to reduced treatment options and increased animal disease and production losses (16). For instance, the World Bank (17) has estimated a 10% production loss in the livestock sector in low- and middle-income countries by 2050. In addition, infected animals may shed these bacteria, posing a threat to other farm animals, household pets, and humans, through direct contact or environmental contamination (11, 18). Infected animals may also act as a reservoir for resistant bacteria, which might enter the food chain (19).

Nowadays, several high-income countries monitor trends in AMU and AMR in livestock (20). These data, however, are generally scarce, particularly from low- and middle-income countries (LMIC) (2). Although access and usage of antimicrobials is improving in LMIC (21), information on actual AMU practices (volume, mode, and reasons for use) is lacking. Specifically, there is a huge gap in the availability of data that can be used to understand the trends over time and to evaluate the linkages between AMU and AMR. The availability of such data can potentially support informed decision-making process especially in connection with the framework of the global action plans formulated by international organizations such as the Food and Agriculture Organization (7), World Organization for Animal Health (9), and WHO (6).

Ethiopia has one of the largest livestock population in Africa with 60.4 million cattle, 31.3 million sheep, 32.7 million goats, and 1.4 million camels (22). Different production systems and agroecological zones coexist, making the process of nationally harmonized guidelines for livestock health and production challenging. This necessitates consideration of representative sampling considering the different agroecological zones of the country in research and development. Similar to many other developing countries, regulations on AMU in livestock in Ethiopia are poorly enforced and farmers have easy access to veterinary drugs; in the worst cases, the drugs may sometimes be falsified or substandard. Moreover, use of drugs in these settings is not commonly supervised by a trained veterinarian. Currently, information regarding AMU in livestock is scarce in Ethiopia, specifically the factors and incentives influencing the use of antimicrobial agents in animals at the farm level are poorly understood. Information on the knowledge, attitudes, and practices (KAP) of farmers regarding antimicrobials and their application will help in formulating strategies to maximize and preserve the benefits of AMU in livestock production with minimal jeopardy to public health. Therefore, we conducted a study to understand knowledge, attitude, and practice of smallholder livestock owners regarding antimicrobial use, resistance and residue in Ethiopia, which can serve as a case study for other comparable production systems.

## Materials and Methods

### Study Area

This study was conducted in three, representative agro-ecological zones and production systems in the Amhara and Oromia regions in Ethiopia: (i) highland mixed crop-livestock production system (Menz Mama and Menz Gera district), (ii) lowland mixed crop-livestock system (Abergelle and Zequwala district), and (iii) pastoral system (Yabello and Eleweya districts).

The highland agroecology with a mixed crop-livestock system is typical for areas above 2,200 m above sea level (masl) and is characterized as a system in which livestock husbandry and rain-fed cropping are closely interlinked. Livestock provide inputs (draft power, transport, and manure) to other parts of the farm system and generate consumable or saleable outputs (milk, meat, eggs, hides and skins, wool, hair, and manure). Crop residues are used as livestock feed; animals can be sold and revenues can be reinvested in agriculture or sold when the crop is failing because of weather or pests; cereals and most staple foods are produced in quantities that cover the needs of the family and excess is sold. The principal objective of farmers engaged in mixed farming is to gain complementary benefit from an optimum mixture of crop and livestock and spreading income and risks over both crop and livestock production (23).

The lowland agroecology with mixed crop-livestock system denotes elevation of ≤1,500 masl where farmers herd livestock in rangelands and produce crops on fertile land. The system is understood in a dual sense: firstly, it refers to farming systems entirely based on livestock but practiced in proximity to and perhaps functional association with cropping farming systems; secondly, it refers to the livestock subsystem of crop-livestock farming.

The lowland agroecology with the pastoral production system is characterized by sparsely populated pastoral rangelands, where subsistence of pastoralists is mainly based on livestock and livestock products. Livestock husbandry in this system is dominated by goats, cattle, sheep, and camels. Since the main source of food is milk, pastoralists tend to keep large herds to ensure sufficient milk supply and generate income by selling dairy products or live animals. The pastoral production system in some areas has been evolving into an agro-pastoral system (24).

### Study Design and Sampling

A cross-sectional study was conducted with 379 smallholder livestock owners in 12 villages in six districts. The agro-ecological zones, districts, and villages were purposively selected to address the representation of different agroecological conditions and production systems. To determine the sample size required for the cross-sectional household survey, the sample size and power calculation tool of Epi InfoTM 7 (CDC, Atlanta, GA) was used. The required sample size of 374 was calculated (assuming allowable error of 6%; design effect of 1.4) and equally distributed to the clusters (agro-ecological zones and production systems). A sampling frame of all households from each of the selected villages was obtained from administration office and 423 households were randomly selected to account for non-participation of the selected households. Finally, the survey was conducted in 379 households. Five households were omitted from the final data analysis due to incomplete information. Each household was visited once.

### Assessment Tool

The antimicrobial use assessment tool was developed and set up in Open Data Kit (ODK) on mobile tablet devices. The tool included open-ended and closed questions about household demographics, farm characteristics, management of manure, feed types, animal health constraints, disease prevention, animal health services, antimicrobial use, animal product consumption, and costs related to animal health. Prior to the study, veterinarians in the localities were trained as enumerators and the questionnaire was piloted with 40 livestock owners as a first step of validating the tool. Each interview took approximately 40 min to complete. Commonly available and used drugs at each study site were bought at the local veterinary drug stores and put in a demonstration box to facilitate interaction of enumerators with livestock keepers in gathering information on which drugs are used on the farm.

### Data Analysis

Descriptive statistics were computed to describe household demographics and farm characteristics. Answers to open questions were coded into categorical variables and analyzed. Chi-square test was used to test potential associations between categorical variables and a *p* < 0.05 was considered as statistically significant.

Twenty-one items were used to assess farmers' knowledge (*n* = 6), attitudes (*n* = 6), and practices (*n* = 9) related to antimicrobial use and resistance. The outcomes concerning knowledge were initially multiple choice or “yes vs. no,” and these were all reclassified as “correct” vs. “incorrect.”

The attitude questions were either “yes vs. no” or on a five-point Likert scale “Strongly disagree” to “Strongly agree.” The five-point Likert scale was grouped as follows: When a respondent indicated “strongly agree” and “agree” with a negative or “undesirable” statement, the response was classified as an “undesirable” attitude. The reverse was considered as a “desirable” attitude. Responses of “neither disagree nor agree” were not included in the analysis.

The response to questions regarding farmer practices were either “yes vs. no” or multiple choice, with the latter being dichotomized as “desirable” vs. “undesirable.” Data were coded by giving 1 to correct or desirable answers and 0 to the wrong or undesirable response to a given question or item.

The percentages of “appropriate” answers (i.e., correct answers in the knowledge section, desirable attitude in the attitude question, and application of appropriate management practices in the practice section) were calculated for each KAP item.

Cronbach's alpha and the item response theory (IRT) model were used to assess the knowledge, attitude, and practice measurements. Internal consistency was evaluated using Cronbach's alpha, a parameter that describes the extent to which all the items in a test measure the same concept and it is thus connected to the inter-relatedness of the items within the test (25).

IRT analysis, which provides information on the discrimination and difficulty of each item across different levels of the underlying trait, was used. IRT is based on the assumption of unidimensionality [there is a single unmeasured (latent) trait underlying all items]. The assumption of unidimensionality was evaluated by subjectively evaluating the eigenvalues and factor loadings derived from an exploratory factor analysis along with an evaluation of relationships among items within a correspondence analysis. Only questions related to practices met the assumption of unidimensionality.

A two-parameter logistic (2PL) model was used for practice items to calculate the probability that a person with a given level of management expertise would implement a specific item. This model is represented by the following equation (26):

$$\begin{array}{l} Pij\;\left(ui=1|\theta =t\right)=1/1+\exp\left[-1.7ai\left(t-bi\right)\right] \end{array}$$

where *a*_*i*_ is the discrimination parameter for item *i* (*i* = 1, …, *n*), *b*_*i*_ is the difficulty parameter for item *i, u*_*i*_ is the response of the person with trait level θ to item *i*, and 1.7 is a scaling constant.

The discrimination parameter is allowed to vary between items. Henceforth, the Item Characteristic Curve (ICC) of the different items can intersect and have different slopes. The steeper the slope, the higher the discrimination of the item, as it will be able to detect subtle differences in the management ability of the respondents. The difficulty parameter reflects how difficult it was for an individual to adopt the appropriate management practice (a high difficulty parameter would indicate that relatively few individuals adopted this practice).

A single composite trait (latent variable) called theta (θ) was used for description or analysis of the ability of person. Predicted values of theta were computed for each respondent based on their aggregate response to the practice questions. Inferential statistics (Mann–Whitney *U*-test) was used to compare the mean values of the predicted thetas across farm and socio-economic characteristics. A *p* < 0.05 was taken as significant for Mann–Whitney *U*-test.

Data was analyzed using Stata software version 14 (Texas, USA).

## Results

### Sociodemographic and Farm Characteristics

Sociodemographic and farm characteristics are summarized in Table 1. Most of the respondents had long experience in keeping livestock but more than half of them reported that they had never been to school (Table 1).

**Table 1:** Household demographics and farm characteristics from a study of antimicrobial use in 374 households in 12 villages in six districts within three agro-ecological zones in Ethiopia.

**Categorical variable**	**Category**	**Highland mixed crop-livestock** **(*****n*** **= 128)**		**Lowland mixed crop-livestock** **(*****n*** **= 126)**		**Mid/lowland pastoral** **(*****n*** **= 120)**		**Total** **(*****n*** **= 374)**	
		***n***	**%**	***n***	**%**	***n***	**%**	***n***	**%**
Sex of the household head	Male	116	90.6	117	92.9	105	87.5	338	90.4
	Female	12	9.4	9	7.1	15	12.5	36	9.6
Sex of respondent	Male	109	85.2	105	83.3	83	69.2	297	79.4
	Female	19	14.8	21	16.7	37	30.8	77	20.6
Age of respondent	≤ 25	18	14.1	8	6.3	25	20.8	51	13.6
	25–55	90	70.3	95	75.4	67	55.8	252	67.4
	≥ 55	20	15.6	23	18.3	28	23.3	71	18.9
Education level	Never went to school	11	5.6	92	24.6	92	24.6	195	52.1
	Primary school	62	53	30	25.6	25	21.4	117	31.3
	Secondary school/College	55	88.7	4	6.5	3	4.8	62	16.6
Illiteracy level	Female	5	26.3	21	0	35	94.5	61	79
	Male	6	5.5	71	67.6	57	68.7	134	45
Type of livestock species	Cattle	128	100	115	91.3	110	91.6	353	94.39
	Sheep	127	99.2	110	87.3	115	95.8	352	94.12
	Goat	21	16.4	124	98.4	117	97.5	262	70.05
	Poultry	122	95.3	65	51.6	79	65.8	266	71.12
	Equine	111	86.7	119	94.4	40	33.3	271	72.45
Livestock species mix	Keep >3 species	116	90.6	100	79.4	86	71.7	302	80.75
	Keep ≤ 3 species	12	9.4	26	20.6	34	28.3	72	19.25
Hired worker on the farm	Yes	4	3.1	54	42.9	1	0.8	59	15.8
	No	124	96.9	72	57.1	119	99.2	315	84.2
Main income source for the household	Crop farming	90	70.3	36	28.6	47	39.2	173	46.3
	Cattle keeping	1	0.8	4	3.2	11	9.2	16	4.3
	Small ruminants	34	26.2	84	66.7	59	49.2	177	47.3
	Other	3	2.3	2	1.6	3	2.5	8	2.1
**GRAZING MANAGEMENT**									
Cattle beef (*n* = 353)	Zero grazing	65	50.5	33	28.7	0	0	98	27.7
	Fenced individual farm grazing	27	21.1	2	1.7	0	0	29	8.2
	Communal grazing	12	9.4	78	67.8	0	0	90	25.5
	Pastoral	0	0	2	1.7	88	80	90	25.5
Cattle dairy (*n* = 353)	Zero grazing	4	3.1	7	6.1	0	0	11	3.1
	Fenced individual farm grazing	69	19.5	3	0.8	0	0	72	20.4
	Communal grazing	55	43	104	90.4	0	0	159	45
	Pastoral	0	0	1	0.9	110	100	111	31.4
Small ruminant (*n* = 371)	Zero grazing	1	0.8	3	2.4	0	0	4	1.1
	Fenced individual farm grazing	63	49.6	3	2.4	0	0	66	17.8
	Communal grazing	63	49.6	118	94.4	1	0.8	182	49.1
	Pastoral	0	0	1	0.8	118	99.2	119	32.1
Poultry (*n* = 266)	Free range	116	95.1	27	41.5	79	100	222	83.5
	Housed	6	4.9	38	58.5	0	0	44	16.5
Equine (*n* = 271)	Zero grazing	0	0	17	15.5	0	0	17	6.3
	Fenced individual farm grazing	62	51.7	3	2.7	0	0	65	24
	Communal grazing	57	47.5	88	80	0	0	145	53.5
	Pastoral	1	0.8	2	1.8	41	100	44	16.2
Sale of milk	Yes	3	2.3	7	5.6	38	31.7	48	12.8
	No	125	97.7	119	94.4	82	68.3	326	87.2
Sale of live animals	Yes	128	100	125	99.2	117	97.5	370	98.9
	No	0	0	1	0.8	3	2.5	4	1.1
Continuous Variable		mean	sd	mean	sd	mean	sd	mean	sd
Size of the household		5.2	1.8	6.3	2.1	7.3	2.8	6.25	2.4
Age of respondent		39.9	12.9	41.7	12.1	40.9	16.4	40.9	13.9
Year of livestock keeping experience		19.9	11.4	20.7	11.1	22.5	15.2	21	12.7
Flock size	Cattle	4.5	1.6	4.9	5.7	15.9	20.5	8.3	13.2
	Sheep	18.8	12.1	16.8	16.1	26.2	39.5	20.5	25.5
	Goat	0.5	1.2	30.6	25.1	32.7	33.5	21	28
	Poultry	5.5	4.9	6.4	4.3	7.3	3.9	6.3	4.6
	Donkey	1.7	0.8	1.5	0.7	1.7	1.6	1.6	0.9

Cattle and sheep were the main livestock species raised by the majority of the respondents (Table 1). The majority of the respondents had mixed type of livestock business with more than three livestock species kept at their farm. Only 16% of the respondents reported to have hired workers on the farm. The main income source for the households was most commonly small ruminant production and crop farming. Most of the respondents reported selling live animals, while sale of milk appeared to be less common. About 50% of respondents reported drinking cow or goat milk at least once per day. Children below 12 years of age were mentioned as the primary milk consumers by the family members in the 69.3% of the surveyed households.

### Animal Diseases and Mortality

Table 2 summarizes the type of reported diseases in the past 12 months. Respiratory diseases were the most commonly mentioned diseases in cattle, sheep, and goat, followed by enteric illnesses. In addition, the proportion of respondents reporting an estimated mortality rate of more than 10% are included in Table 2.

**Table 2:** Owner reported occurrence of animal diseases from a total of 374 households in Ethiopia.

	**Cattle** **(*****n*** **= 350)**		**Sheep** **(*****n*** **= 352)**		**Goat** **(*****n*** **= 262)**	
**Disease**	***n***	**%**	***n***	**%**	***n***	**%**
Respiratory diseases	95	26.9	147	41.7	100	38.2
Digestive tract/enteric illnesses	57	16.2	86	24.4	84	32.1
Reproductive diseases	2	0.6	5	1.42	5	1.9
Sudden death	6	1.7	8	2.3	5	1.9
Skin disease	17	4.8	2	0.6	1	0.4
Gastro-intestinal parasites	8	2.3	2	0.6	0	0
Neurological	0	0	17	4.9	20	7.6
Systemic disease	17	4.9	0	0	0	0
Other	16	4.57	11	3.1	9	3.44
No disease	132	37.4	73	20.7	38	14.5
Mortality >10%	34	9.7	131	37.2	121	46.2

### Drug Use

From the livestock species present, livestock owners used drugs mostly for sheep, cattle, and goats. There was a difference in the type of drugs used between agro-ecological zones and production systems (Table 3). In the highland mixed crop livestock system, the most frequently reported use drugs were anthelmintics (95%), antibiotics (24%), and acaricides (4.7%). Pastoralists mostly used antibiotics (86.7%) followed by anthelmintics (70.8%) (Table 3). The proportion of anthelmintics usage was higher in highland mixed crop-livestock and pastoral than in the lowland crop-livestock system. The use of acaricides was less common compared to the use of other drugs in all agroecologies and production systems studied. Moreover, only 13% of the pastoralists did not have any antibiotic at hand during the survey. Drugs at hand were mostly stored under suboptimal conditions and exposed to change of temperature, sunlight, and dust. Human preparation antibiotics (tetracyclines) were also being used for veterinary purposes by 18.5% of pastoralist households, indicating high level of crossover use. Overall, tetracyclines (36.4%), aminoglycosides (31.3%), and trimethoprim-sulfonamides (6.2%) were the most frequently used classes of antibiotics across the study sites. Benzimidazoles (49.5%) were the most frequently used anthelmintic drugs followed by macrocyclic lactones (29.9%) and triclabendazole (24.6%). Triclabendazole and fenbendazole were only reported from highland mixed crop-livestock systems (Table 4).

**Table 3:** Self-reported antimicrobial use from a total of 374 households in 3 agro-ecological zones in Ethiopia.

	**Highland mixed crop-livestock (*****n*** **= 128)**		**Lowland mixed crop-livestock (*****n*** **= 126)**		**Mid/lowland pastoral** **(*****n*** **= 120)**		**Total (*****n*** **= 374)**	
	**Freq**	**%**	**Freq**	**%**	**Freq**	**%**	**Freq**	**%**
Antibiotics	31_a_	24.2	29_a_	23	104_b_	86.7	164	43.9
Anthelminthics	122_a_	95.3	40_b_	31.6	85_c_	70.8	247	66
Acaricides	4_a_	3.1	1_a_	0.8	36_b_	30	41	10.9

*Each subscript letter denotes a subset of agro-ecological zones whose column frequency does not differ significantly from each other at the 0.05 level*.

**Table 4:** Common antimicrobial groups used by farmers from a total of 374 households in three agro-ecological zones in Ethiopia.

	**Highland mixed crop-livestock (*****n*** **= 128)**		**Lowland mixed crop-livestock (*****n*** **= 126)**		**Mid/lowland pastoral** **(*****n*** **= 120)**		**Total (*****n*** **= 374)**	
	***n***	**%**	***n***	**%**	***n***	**%**	***n***	**%**
**Classes of antibiotics**								
Tetracyclines	20_a_	15.6	26_a_	20.6	90_b_	75	136	36.4
Trimethoprim-sulfonamides	3_a_	2.34	1_a_	0.79	20_b_	16.67	24	6.24
Penicillins	0_a_	0	0_a_	0	5_b_	4.17	5	1.34
Macrolides	0_a_	0	0_a_	0	17_b_	14.17	17	4.55
Aminoglycosides	18_a_	14.1	3_b_	2.4	96_c_	80	117	31.3
**Groups of antihelimintics**								
Albendazole/benzimidazole	84_a_	65.6	40_b_	31.8	61_c_	50.8	185	49.5
Triclabendazole	92_a_	71.9	0_b_	0	0_b_	0	92	24.6
Fenbendazole	6_a_	4.69	0_b_	0	0_b_	0	6	1.6
Ivermectin (Macrocyclic lactones)	37_a_	28.9	1_b_	0.79	74_c_	61.7	112	29.9
Imidazothiazole (Tetramizole, Tetraclozan, Clozasole)	65_a_	50.8	1_b_	0.8	0_b_	0	66	17.6

*Each subscript letter denotes a subset of agro-ecological zones whose column frequency does not differ significantly from each other at the 0.05 level*.

#### Reasons for Use of Antimicrobials

**Figure 1:**
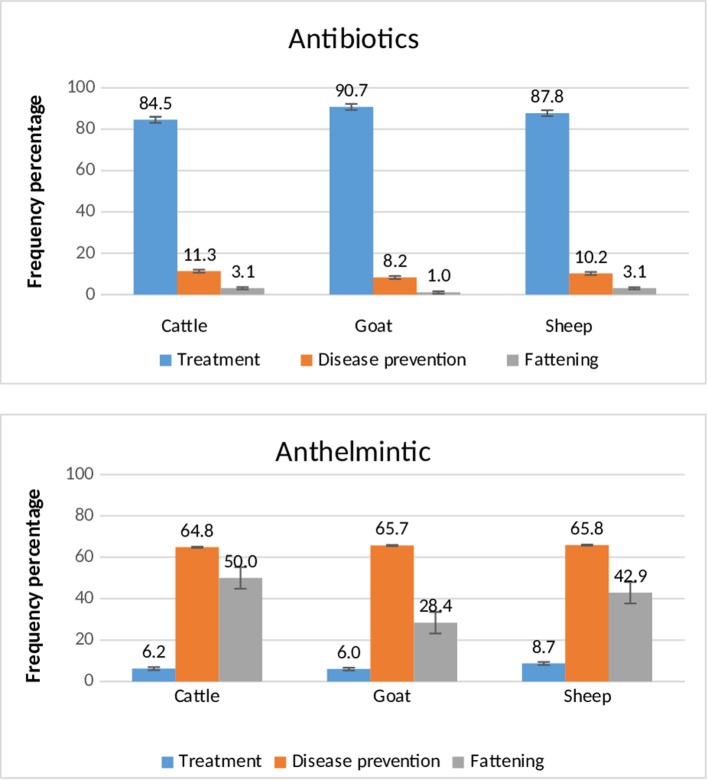
Reason for the use of antibiotics and anthelmintics in different species reported by livestock owners from 374 households in 3 agro-ecological zones in Ethiopia (Frequency percentage and standard error bars).

Use of antimicrobials for prophylactic purposes was common. For the most frequently used drugs over the 12 months prior to the survey, antibiotics were mainly used for treatment purposes, whereas anthelmintics were used for disease prevention and livestock fattening purposes (Figure 1). Respiratory diseases and digestive/internal parasitic infections were the main reasons for therapeutic use of antimicrobials.

#### Access and Source of Veterinary Drugs

**Figure 2:**
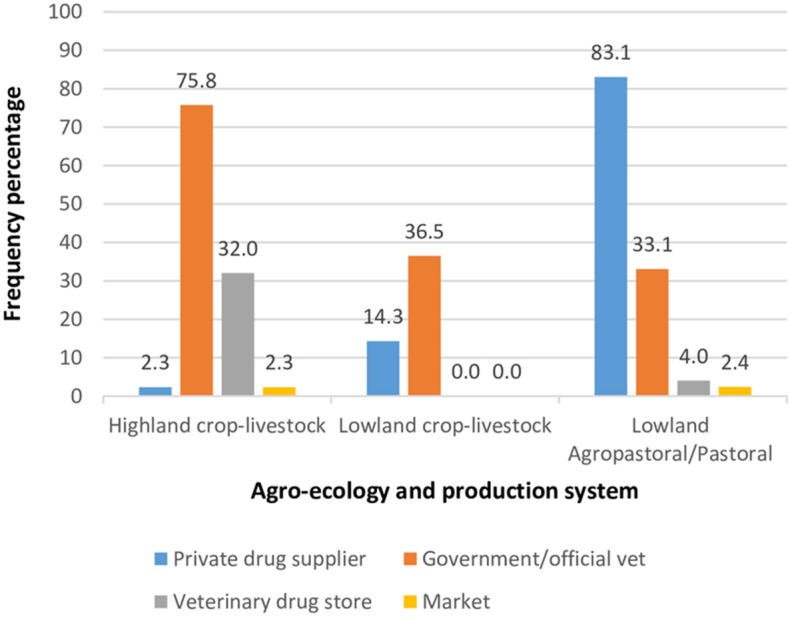
Source of veterinary drugs used by 374 households in 3 agro-ecological zones and production systems in Ethiopia.

Overall, about 81.6% of livestock owners surveyed had access to veterinary drugs, although access varied between agro-ecological zones and production systems. Farmers in the highland mixed crop-livestock systems and the lowland pastoral systems reported access to veterinary drugs (97.7 and 93.3%, respectively), while the corresponding figure for livestock owners in the lowland mixed crop-livestock systems was 54%. The main source of veterinary drugs for livestock owners in both the highland and lowland mixed crop-livestock systems was the government or official veterinarian, whereas pastoralists most commonly accessed drugs from private suppliers (Figure 2).

#### Source of Information and Advice

Almost all respondents in the highland (99%) and 82% of respondents in the lowland mixed crop-livestock systems revealed that they received information and advice on veterinary drug use from a range of sources: veterinarians and animal health workers (78.3% of respondents), drug stores (9.4%), markets (2.7%), and other farmers (8.6%). Among the pastoralists, 74% reported not to depend on any of these sources and reported to commonly decide based on their own judgement on the kind of drugs to use, dose, and treatment duration.

#### KAP Related to Antimicrobial Use, Resistance, and Residue

Regarding the knowledge about antibiotic use, 84.2% of respondents were well aware that antibiotics are useful for treating and preventing infections. However, more than 50% of the respondents had inadequate understanding of antibiotics and they thought antibiotics could help to treat any kind of diseases, regardless of the cause. Moreover, a relatively high proportion of the respondents (>70%) were not aware of the recommended withdrawal periods of milk and meat after antibiotic treatment. Only 20% of livestock owners reported to have heard about antimicrobial resistance and at least 12% mentioned that they had experienced situations where drugs did not work.

About 82% of the respondents were aware that vaccines are generally administered as a preventive measure against infections. There was variation in livestock owners' knowledge of antibiotics between the different agro-ecological zones and production systems (Table 5).

**Table 5:** Knowledge about antibiotic use, resistance and residue (*n* = 374).

**Questions**	**Levels**	**Responses**	**Highland crop-livestock** **(*****n*** **= 128)**		**Lowland crop-livestock** **(*****n*** **= 126)**		**Mid/lowland pastoral (*****n*** **= 120)**		**Overall**	
			**freq**	**%**	**freq**	**%**	**freq**	**%**	**freq**	**%**
K1_What does vaccination do?	Correct	Prevent animals from becoming sick	108_a_	84.4	102_a_	80.9	100_a_	83.3	310	82.9
	Incorrect		20	15.6	24	19.1	20	16.7	64	17.1
K2_What do antibiotics do?	Correct	Cure sick animals and prevent animals from becoming sick	89_a_	69.5	114_b_	90.5	112_b_	93.3	315	84.2
	Incorrect		39	30.5	12	9.5	8	6.0.7	59	15.8
K3_For how long should milk be avoided (in days) immediately after treatment of animals with antibiotics?	Correct	7–30 days depending on the label, as advised	81_a_	63.3	20_b_	15.9	2_c_	1.7	103	27.5
	Incorrect		47	36.7	106	84.1	118	98.3	271	72.5
K4_For how long should meat be avoided (in days) immediately after treatment of animals with antibiotics?	Correct	7–30 days depending on the label, as advised	68_a_	53.1	40_b_	31.8	0_c_	0	108	28.9
	Incorrect		60	46.8	86	68.2	120	100	266	71.1
K5_Have you ever heard about antimicrobial resistance?	Correct	Yes	38_a_	29.7	14_b_	11.1	23_a, b_	19.2	75	20.1
	Incorrect	No	90	70.3	112	88.9	97	80.8	299	79.9
K6_Antibiotics help treat any kind of diseases.	Correct	No	95_a_	74.2	46_b_	36.5	43_b_	35.8	184	49.2
	Incorrect	Yes	33	25.8	80	63.5	77	64.2	190	50.8

*Each subscript letter denotes a subset of agro-ecological zones whose column frequency does not differ significantly from each other at the 0.05 level*.

Regarding the attitudes and perceptions related to antimicrobial use, around 50% stated that they would use antimicrobials more often if antimicrobials were more accessible and cheaper. It was noticed that around 80 and 70% of respondents had a tendency to use doses that were higher or lower than recommended for their animals during treatment, respectively.

About 69% were of the opinion that once the animal started to recover, there was no need to continue giving the full treatment course. Around 21.7% of the respondents had a tendency of keeping leftover antimicrobials at home, as they might be useful in the future (Table 6).

**Table 6:** Attitudes and perceptions on antimicrobial use, resistance and residues (*n* = 374).

**Questions**	**Levels**	**Responses**	**Highland crop-livestock** **(*****n*** **= 128)**		**Lowland crop-livestock** **(*****n*** **= 126)**		**Mid/lowland pastoral (*****n*** **= 124)**		**Overall**	
			**freq**	**%**	**freq**	**%**	**freq**	**%**	**freq**	**%**
A1_Is consuming milk or meat from animals who were just treated with antimicrobials good for human health?	Undesirable	Yes	2	1.6	12	10.3	8	7	22	6.2
	Desirable	No	125	98.4	104	89.7	106	93	335	93.8
A2_If antimicrobials were more accessible and at a lower price, would you use antimicrobials more often?	Desirable	No	67	52.3	79	62.7	44	36.7	190	50.8
	Undesirable	Yes	61	47.7	47	37.3	76	63.3	184	49.2
A3_To get a better response, I sometimes give more antimicrobials to animals than the dose advised by the veterinary clinician or pharmacist.	Desirable	Strongly disagree, disagree	8	6.3	49	41.2	14	12.8	71	20
	Undesirable	Strongly agree, agree	119	93.7	70	58.8	95	87.2	284	80
A4_It is advisable to always reduce the amount/dose of antimicrobial advised by veterinary clinician to avoid harming animals.	Desirable	Strongly disagree, disagree	9	7.1	76	63.9	19	17.8	104	29.5
	Undesirable	Strongly agree, agree	118	92.9	43	36.1	88	82.2	249	70.5
A5_Once the animal starts to feel better, there is no need to continue giving the full dose.	Desirable	Strongly disagree, disagree	8	6.3	21	17.2	81	75	110	30.8
	Undesirable	Strongly agree, agree	119	93.7	101	82.8	27	25	247	69.2
A6_I normally keep leftover antimicrobials for a long time at home because they might be useful in the future.	Desirable	Strongly disagree, disagree	18	14.1	24	20	35	33	77	21.7
	Undesirable	Strongly agree, agree	110	85.9	96	80	71	67	277	78.3

Regarding practices related to antimicrobial use (Table 7), a large proportion of the respondents reported that they commonly consumed milk (36.4%) and meat (51.8%) from animals that had just been treated with antimicrobials, although they assumed it might not be good for human health. The majority of pastoralists (88.6% consumed milk and 98.3% consumed meat) reported this practice.

**Table 7:** Antibiotic use and related practices (*n* = 374).

**Questions**	**Levels**	**Responses**	**Highland crop-livestock** **(*****n*** **= 128)**		**Lowland crop-livestock** **(*****n*** **= 126)**		**Mid/lowland pastoral (*****n*** **= 124)**		**Overall**	
			**freq**	**%**	**freq**	**%**	**freq**	**%**	**freq**	**%**
P1_Do you consume milk from animals who were just treated with antimicrobials?	Desirable	No	115	90.5	99	85.3	13	11.4	227	63.6
	Undesirable	Yes	12	9.5	17	14.7	101	88.6	130	36.4
P2_Do you consume meat from animals who were just treated with antimicrobials?	Desirable	No	102	80.3	68	58.6	2	1.8	172	48.2
	Undesirable	Yes	25	19.7	48	41.4	112	98.3	185	51.8
P3_How long do you use antibiotics in animals?	Desirable	As advised	127	100	99	79.2	33	27.7	259	69.8
	Undesirable	Until animal(s) cured; Until package empty; As long as I can afford; One time treatment; Continuously over extended period	0	0	26	20.8	86	72.3	112	30.2
P4_What do you do with expired veterinary drugs?	Desirable	Dispose of, Return to pharmacy; don't receive	117	91.4	56	45.5	118	98.3	291	78.4
	Undesirable	Give to other farmer; Use for intended treatment; Nothing	11	8.6	67	54.47	2	1.67	80	21.6
P5_How do you manage manure?	Desirable	Used as fertilizer; Use for fuel (incl. biogas); Sold for cash (fuel)	126	99.2	125	100	1	0.8	252	67.9
	Undesirable	Leave on farm; Open air; Discard into environment	1	0.8	0	0	118	99.2	119	32.1
P6_Do you have isolation pen for sick animals?	Desirable	Yes	87	68	72	57.1	21	17.5	180	48.1
	Undesirable	No	41	32	54	42.9	99	82.5	194	51.9
P7_Do you allow animals on treatment to immediately freely graze with other animals without quarantine for few days?	Desirable	No	97	75.8	66	52.4	62	51.7	225	60.2
	Undesirable	Yes	31	24.2	60	47.6	58	48.3	149	39.8
P8_What do you do if an animal dies from disease?	Desirable	Bury, burn	25	19.5	7	5.6	1	0.8	33	8.8
	Undesirable	Leave as it is; give to the dog; home consumption	103	80.5	119	94.4	119	99.2	341	91.2
P9_Who administers the antibiotics?	Desirable	Veterinarian; animal health practitioners	128	100	124	98.4	0	0	252	67.4
	Undesirable	Myself	0	0	2	1.6	120	100	122	32.6

Overall, the majority of the respondents (70%) administered antibiotics as advised, but 72.3% of pastoralists administered antibiotics by not following through the full treatment course: “until the animal cured,” “until package empty,” “as long as they can afford,” “one time treatment or continuously over extended period.” All pastoralists self-administered antibiotics to their animals without any laboratory diagnosis. About 98% of pastoralists had good practice with regard to care of expired veterinary drugs, which they either disposed of by burying or returning to the vendor. Indeed, during data collection, 97% of the pastoralist households did not have any expired antimicrobial at hand.

Half of the respondents (50%) reported to have an isolation pen for sick animals and 40% indicated that they would allow animals currently receiving treatment to immediately freely graze with other animals without quarantine. Only 9% of the respondents implemented proper practices regarding disposal of dead animals, either through burial or incineration. The majority (97.5%) of the pastoralists and 4% of respondents from each of the highland and lowland mixed crop production systems revealed consumption of dead animals.

### Assessment of the KAP Measurement Scales

Cronbach's alphas were poor for the knowledge (0.478) and attitude (0.319) scales, and the inter-item correlations were low. But the Cronbach's alpha was high for practice scale (0.816). “P4” from the practice scale presented a negative biserial coefficient and was therefore excluded from further analyses. The factor and correspondence analysis suggested that the knowledge and attitude scales were not unidimensional, and consequently, these scales were not used to develop IRT models. Based on a factor analysis of the practice scale, the assumption of unidimensionality seemed to be met. The first eigenvalue was 15 times larger than the second and accounted for 97% of the total variation.

The discrimination (*a*_*i*_) and difficulty (*b*_*i*_) parameters from the IRT analysis of the practice scale are presented in Table 8.

**Table 8:** Discrimination and difficulty values of the items in the practice scale (sorted by decreasing discrimination).

	**Items**	**Coef**.	**Std. Err**.	**95% Conf. Interval**	
Discrimination	P5_How do you manage manure?	4.53	0.58	3.379	5.685
	P9_Who administers the antibiotics?	4.48	0.58	3.334	5.685
	P1_Do you consume milk from animals who were just treated with antimicrobials?	3.51	0.51	2.492	4.52
	P2_Do you consume meat from animals who were just treated with antimicrobials?	3.18	0.49	2.196	4.154
	P3_How long do you use antibiotics in animals?	2.59	0.38	1.844	3.342
	P6_Do you have isolation pen for sick animals?	1.69	0.27	1.158	2.23
	P8_What do you do if an animal dies from disease?	1.67	0.92	−0.146	3.484
	P7_Do you allow animals on treatment to immediately freely graze with other animals without quarantine for few days?	0.59	0.12	0.344	0.853
Difficulty	P5_How do you manage manure?	−0.47	0.07	−0.611	−0.321
	P9_Who administers the antibiotics?	−0.45	0.07	−0.598	−0.308
	P1_Do you consume milk from animals who were just treated with antimicrobials?	−0.32	0.08	−0.478	−0.171
	P2_Do you consume meat from animals who were just treated with antimicrobials?	0.21	0.08	0.056	0.37
	P3_How long do you use antibiotics in animals?	−0.63	0.09	−0.814	−0.469
	P6_Do you have isolation pen for sick animals?	0.2	0.09	0.0153	0.396
	P8_What do you do if an animal dies from disease?	2.59	0.69	1.248	3,946
	P7_Do you allow animals on treatment to immediately freely graze with other animals without quarantine for few days?	−0.63	0.23	−1.092	−0.184

**Figure 3:**
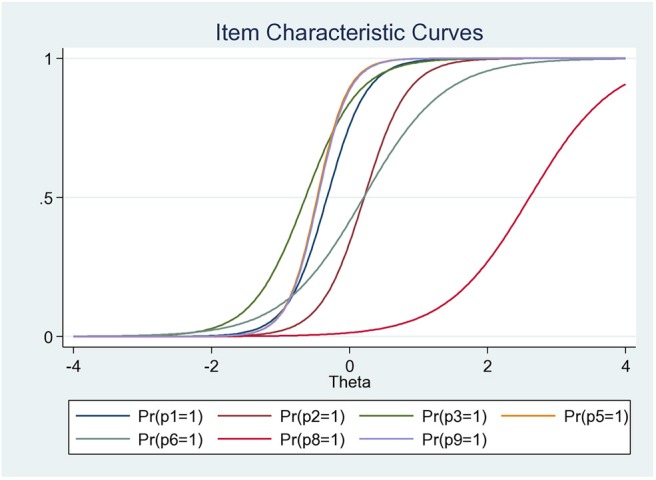
Item characteristic curve for the 7 items used make up the scale related to antibiotics use practices.

**Figure 4:**
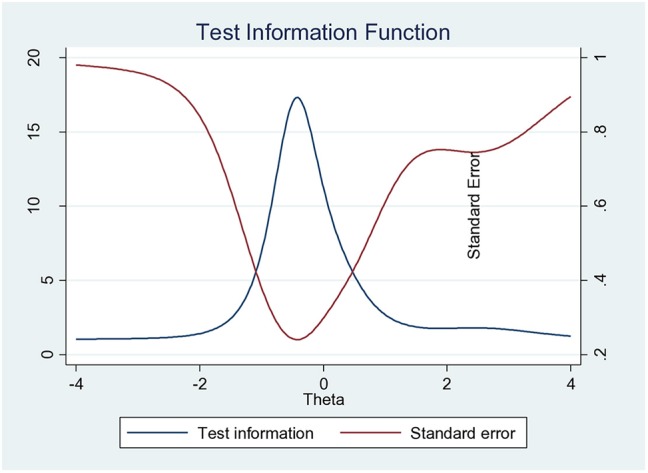
Test information curve for the scale related to antibiotic use practices.

Most of the practice items have a similar discrimination level and a similar low level of difficulty except for the item “P8” with higher difficulty (*b*_*i*_ = 2.59), but low discrimination (*a*_*i*_ = 1.66). Items “P5” (*a*_*i*_ = 4.53) and “P9” (*a*_*i*_ = 4.47) had relatively high discrimination power, whereas “P7” had very low discrimination (*a*_*i*_ =0.59), suggesting that it contributed little to the scale (Table 8). On the basis of all this information, it appears that we can make a useful, unidimensional seven-item scale (P1, P2, P3, P5, P6, P8, and P9) (Figure 3). Therefore, the scale was able to differentiate among people with a level management expertise of theta between −1.5 to 1 (Figure 4), respectively, answering between 0 and 7 questions correctly.

### Association of Household Demographics and Farm Characteristics, With Desirable Practices

A single composite trait or variable called theta (θ) was used to characterize the ability of person to perform desirable practices instead of a descriptive summative scale for practice. The composite variable provides an overall estimate of the quality being measured (management ability of person). It takes into account the difficult and discrimination values for each item and hence is a more reliable overall measure than a simple sum of the individual items in the scale. For each respondent, a theta (θ) score was computed and the mean theta of different groups, based on farm and socio-economic characteristics, were compared. Higher means indicated better desirable practice in a specific group of respondents (Table 9).

**Table 9:** Comparison of household demographics and farm characteristics and ability to give desirable response for practice questions.

**Description**	***N* (374)**	**Theta** **Mean (SE)**
**Agroecology and production system**		
Highland mixed crop-livestock production system	128	0.84 (0.04)_a_
Lowland mixed crop-livestock production system	126	0.45 (0.04)_b_
Pastoral/agro-pastoral production system	120	−1.15 (0.02)_c_
**Sex of respondent**		
Male	297	0.13 (0.05)_a_
Female	77	−0.14 (0.11)_a_
**Education**		
Never went to school	195	−0.28 (0.06)_a_
Primary school	117	0.28 (0.08)_b_
Secondary school/College	62	0.78 (0.08)_c_
**Age**		
Young (<30)	84	−0.04 (0.10)_a_
Medium (30–50)	204	0.13 (0.06)_a_
Old (>50)	86	0.02 (0.10)_a_
**Livestock experience**		
< = 5 year	30	0.19 (0.18)_a_
5–20	184	0.02 (0.07)_a_
>20 year	160	0.10 (0.07)_a_
**Household size**		
Small (<4 person)	37	0.46 (0.15)_a_
Medium (4–8 person)	279	0.14 (0.05)_a_
Large (>8 person)	58	−0.55 (0.12)_b_
**Species mix**		
3 and less species	72	−0.29 (0.11)_a_
More than 3 species	302	0.16 (0.05)_b_
**Hired worker**		
Yes	59	0.62 (0.06)_a_
No	315	−0.03 (0.05)_b_

*Each subscript letter denotes a subset of agro-ecological zones whose column frequency does not differ significantly from each other at the 0.05 level (Mann–Whitney test)*.

Among the variables, there were significant differences in the mean theta for agro-ecology/production system, education level, having hired workers on the farm, having more than 3 different livestock species, and household size (*p* < 0.05) (Table 9). Respondents from highland mixed crop livestock production system had a higher mean for theta than those of lowland mixed crop livestock and pastoral production system. However, there were no significant differences in the mean theta according to age group of the respondents or their livestock keeping experience.

## Discussion

Antimicrobial resistance (AMR) has been recognized as a global health problem. Monitoring of antimicrobial use (AMU) provides useful information for policy development to mitigate AMR risks and therefore has been recommended by international organizations (6, 7, 9).

In Ethiopia, like other sub-Saharan countries, it is generally believed that antimicrobial agents are widely used in animal production systems; however, evidence on antimicrobial usage is limited and often anecdotal. We found only a single survey that evaluated the rational use of veterinary drugs, and it focused only on the college of veterinary medicine and agriculture veterinary teaching hospital and Ada district veterinary clinic of central Ethiopia (27).

This study characterized antimicrobial (includes anthelmintic) use knowledge, attitude, and practice in smallholder settings in three different agro-ecology and production system. To our knowledge, this study is the first to investigate antimicrobial usage in livestock by smallholder farmers and pastoralists in Ethiopia. Most of the respondents were adults with many years of experience in keeping livestock.

We found that the use of antimicrobial agents in livestock production was very common among the livestock producers in the study areas. Antimicrobial use may vary widely between and within countries, species, production systems, and individual farms (28). This is also what we found in our study. The data on use of antimicrobial agents were not restricted to any particular livestock species but cut across mainly three livestock species (cattle, sheep, and goat) and equine and poultry in few cases. We observed large variation in the choice of drugs and proportion of respondents who had used antimicrobials among smallholder farmers in the three agro-ecology and production systems included in the study.

Livestock producers in mid/lowland pastoral systems appeared to use antibiotics more frequently than their counterparts in highland and lowland mixed crop-livestock systems. Tetracyclines, aminoglycosides, and trimethoprim-sulfonamides were the most dominantly used classes of antibiotics. Penicillins and macrolides were only reported to be used by the pastoral production systems. This is consistent with studies elsewhere that reported these antimicrobials to be frequently used in food animals in Africa (16, 27, 29–33). The penicillin, tetracycline, and aminoglycoside classes were also the most commonly reported antimicrobial usages across pig production systems in Thailand and Vietnam (34).

Despite known deficits in animal health services in Ethiopia, the livestock owners had good access to veterinary drugs. The main source of veterinary drugs in both the highland and lowland mixed crop-livestock systems was the government or official veterinarians, whereas the pastoralists most commonly accessed drugs from private suppliers. The study found that farmers tended to give higher or lower doses of antimicrobials to their animals than recommended. Medically irrational use of antimicrobials in food animals is known to contribute to the emergence, persistence, and spread of resistant bacteria from animals to humans (7). Regarding information and advice on antimicrobial usage in livestock, this study found that a high proportion of the pastoralists rely on their own judgment. Hence, it was not surprising that we found high levels of potentially wrong use of antibiotics. Access to antimicrobials without prescriptions results in increased risk for antimicrobial resistant pathogens, which has also been shown elsewhere in Africa (16, 30).

The inappropriate antimicrobial use by pastoralists might be linked with this ease of access and inadequate advice for farmers (35). Restricting access to antimicrobials by removing over-the-counter sales has been identified as a potential route to better antimicrobial use in animals (6, 36).

Moreover, the reported frequent use of cow or goat milk in their meal coupled with a relatively high proportion of farmers not being aware of the recommended withdrawal periods of milk and meat after antibiotic treatment may lead to the potential hazard of repeatedly ingested residues altering the intestinal microbiome and promoting emergence and selection for resistant bacteria in the gastrointestinal tract of humans (37, 38). Withdrawal times are recommended in order to prevent the presence of drug residues in food products (39).

There is a possible risk of an infectious disease being transmitted from animals to human due to a habit of consumption of dead animals. The poor experience of isolating sick animals and improper disposal of dead animals by the majority of the farmers in this study illustrates the negligence of biosecurity practices and other precautionary measures to prevent infectious agents. However, infection prevention and control measures are crucial in order to reduce the incidence of infections and, therefore, reduce the need for antibiotics (40–42). Besides, the non-involvement of laboratory investigations in disease diagnosis prior to antimicrobial further fuels inappropriate use of antimicrobials, which may subsequently lead to the development and spread of AMR (43, 44), which definitely is a big challenge in Ethiopia.

Despite the frequent use of antimicrobials by smallholder farmers to maintain good livestock health and production in the studied areas, there was overall poor knowledge about the purpose of antibiotics and their proper use. Poor knowledge may be the result of the fact that more than half of the smallholder farmers never went to school or have otherwise poor education. Farmers generally thought antibiotics could help treat any kind of diseases regardless of the causes. This could result in inappropriate antibiotic use with potential risks of antibiotic-resistant pathogens that will lead to treatment failures, increased mortality and production losses, and also possible human health risks (45). Founou et al. (46) also indicated that 86.6% of multidrug-resistant bacteria were detected in food animals at farms in Africa, which may be indicative of widespread use of antibiotics in farming practices, whereas 52.4% detected at abattoirs reflected bacteria surviving the processing stage and, therefore, able to reach the consumer.

Another finding of our study was the difference in the ability of respondents to give desirable response for practice on the basis of the agro-ecology and production system. Respondents from highland mixed crop livestock production systems were more likely to have higher ability to give desirable response for practice questions than those in lowland mixed crop livestock and pastoral production systems. There was a link between better ability of a person to perform desirable practices and higher education level. Besides the education barrier, limited professional supervision can also have an impact on a farmer's practice as reflected in the pastoral production system. Wrong public perception, attitudes, and beliefs about antibiotics are strong determinants of medically irrational use of antibiotics (43). It has been suggested that increasing knowledge and awareness about antibiotics and antibiotic resistance are key components of rational antibiotic use in human medicine (6, 7). While improving the knowledge and attitudes of smallholders can encourage them to practice medically rational use of antimicrobials, addressing the drivers for use is as important to achieve lasting behavior change.

IRT methods allow researchers to improve measurement scale construction and evaluate the quality of individual items. In this study, the 2PL logistic models fitted practice scales reasonably well. The Cronbach's alpha for knowledge and attitude scales was low, reflecting that the items were not internally consistent. But the corresponding value yielded for practice was high. The likely reason for low reliability may be that knowledge and attitude measurement items cover different dimensions like purpose of antimicrobial use, disease prevention, antimicrobial residue, and biosecurity issues and evaluate different concepts. The implications of these findings are that future research should focus on assessment of more extensive knowledge and attitude measurement scales toward AMU, AMR, and residues. Approaches that identify the quality of individual items that specifically measure one thing at a time for the knowledge and attitude scale construction should be attempted. The focus should be on the coverage of the content the instrument is supposed to measure. It is also necessary to include new items with high discrimination of knowledge and attitude and greater accuracy of measurement.

Findings of this study help to target future interventions to reduce antimicrobial use and resistance in the smallholder livestock systems of Ethiopia. While it is impossible to extrapolate data from this study to other sub-Saharan African countries, tools and methods used here can easily be applied elsewhere.

## Limitation of the Study

Though the study was piloted with 40 participants, there was no instrument to objectively assess the honesty and recall ability of the participants. The training of enumerators on data collection and use of a demonstration box with drugs to facilitate the enumerator in gathering antimicrobial usage information helped to reduce this possible bias. In addition, as with most surveys, there is the possibility of social desirability bias that respondents may be over- or underreporting antimicrobial use.

The scale used to assess the knowledge and attitudes regarding antimicrobial use consists of a mix of different topics like disease prevention, drug residue, and biosecurity. The number of questions was also minimized with the intention of reducing the time taken to complete the questionnaire.

## Data Availability Statement

The datasets generated for this study are available on request to the corresponding author.

## Ethics Statement

Ethics approval (Certificate Ref. No: VM/ERC/01/07/10/2018) was obtained from the Addis Ababa University, College of Veterinary Medicine and Agriculture Animal Research Ethics Review Committee, and the Institutional Research Ethics Committee of the International Livestock Research Institute (ILRI-IREC2018-24). Written informed consent for participation was not required for this study in accordance with the national legislation and the institutional requirements.

## Author Contributions

BG, BW, KA, and UM conceived and designed the study. BG, BW, HD, and GA followed up and monitored data collection. BG, ID, and GH analyzed the data. BG, BW, ID, GH, and KA conceptualized and drafted the paper. All authors read, commented on, and approved the final manuscript.

### Conflict of Interest

The authors declare that the research was conducted in the absence of any commercial or financial relationships that could be construed as a potential conflict of interest.
